# *In Silico* Characterization of Histidine Acid Phytase Sequences

**DOI:** 10.1155/2012/845465

**Published:** 2012-12-05

**Authors:** Vinod Kumar, Gopal Singh, A. K. Verma, Sanjeev Agrawal

**Affiliations:** ^1^Department of Biochemistry, G. B. Pant University of Agriculture & Technology, Pantnagar 263145, India; ^2^Akal School of Biotechnology, Eternal University, Baru Sahib, Sirmour 173101, India

## Abstract

Histidine acid phytases (HAPhy) are widely distributed enzymes among bacteria, fungi, plants, and some animal tissues. They have a significant role as an animal feed enzyme and in the solubilization of insoluble phosphates and minerals present in the form of phytic acid complex. A set of 50 reference protein sequences representing HAPhy were retrieved from NCBI protein database and characterized for various biochemical properties, multiple sequence alignment (MSA), homology search, phylogenetic analysis, motifs, and superfamily search. MSA using MEGA5 revealed the presence of conserved sequences at N-terminal “RHGXRXP” and C-terminal “HD.” Phylogenetic tree analysis indicates the presence of three clusters representing different HAPhy, that is, PhyA, PhyB, and AppA. Analysis of 10 commonly distributed motifs in the sequences indicates the presence of signature sequence for each class. Motif 1 “SPFCDLFTHEEWIQYDYLQSLGKYYGYGAGNPLGPAQGIGF” was present in 38 protein sequences representing clusters 1 (PhyA) and 2 (PhyB). Cluster 3 (AppA) contains motif 9 “KKGCPQSGQVAIIADVDERTRKTGEAFAAGLAPDCAITVHTQADTSSPDP” as a signature sequence. All sequences belong to histidine acid phosphatase family as resulted from superfamily search. No conserved sequence representing 3- or 6-phytase could be identified using multiple sequence alignment. This *in silico* analysis might contribute in the classification and future genetic engineering of this most diverse class of phytase.

## 1. Introduction

Phytate (*myo*-inositol 1,2,3,4,5,6-hexa*kis*phosphate; IP_6_) is the major storage form of phosphorus (P), representing approximately 80% of P in soil [1], 65–80% of total P in grains [2], and up to 80% of P in manures from monogastric animals [3]. Phytate exists primarily as metal phytate complex with nutritionally important cations, that is, Ca^2+^, Fe^2+^, and Zn^2+^ [4]. 

Phytases (IP_6_ phosphohydrolase) are a class of phosphatases which catalyses hydrolysis of phytate to inositol phosphates, inorganic phosphorus, and *myo*-inositol [5], also lowers down affinity of phytate to associated minerals and proteins [6], and thus increases bioavailability of P, minerals, and proteins for growth and development of plants and animals [7–9].

Phytases are widely distributed among plants [10, 11], certain animal tissues, and microbial cells [12–15]. To date, four classes of phytases have been characterized in terrestrial organisms: histidine acid phytase (HAPhy), cysteine phytase (CPhy), purple acid phosphatase (PAP), and *β*-propeller phytase (BPPhy) [16, 17]. HAPhys are the most studied and diverse class of phytase. Most bacterial, fungal, and plant phytases belong to histidine acid phosphatases (EC 3.1.3.2) which are further classified as 3-phytase (EC 3.1.3.8) or 6-phytase (EC 3.1.3.26) due to their high specific activity for phytate and position specific initial hydrolysis of phytate. 

Phytases have been extensively reviewed for various industrial and biotechnological applications [18–21], biochemical properties [22], and consensus phytase construct [23]. Conserved amino acid residues are reported in HAPhy sequences at N-terminal “RHGXRXP,” C-terminal “HD,” and eight cysteine residues in around sequence [16, 24, 25]. It is a well-adopted fact that all phytases have not similar and common active site; hence the initial classification system is based on catalytic mechanism [22]. Still, there is a need to devise a taxonomic system to accommodate new types of phytases with novel catalytic mechanism. 

The *in silico* characterization of protein sequences of industrially important enzymes has been reported recently [26–28]. Biochemical features, homology search, multiple sequence alignment, phylogenetic tree construction, motif, and superfamily distribution of alkaline proteases have been analyzed using various bioinformatics tools [28]. A total of 121 protein sequences of pectate lyases were subjected to homology search, multiple sequence alignment, phylogenetic tree construction, and motif analysis [26]. Malviya et al. [27] collected forty-seven full-length amino acid sequences of PPO from bacteria, fungi, and plants and subjected them to multiple sequence alignment (MSA), domain identification, and phylogenetic tree construction.

In the present study, we performed *in silico* analysis of 50 HAPhy protein sequences. The biochemical features, homology search, multiple sequence alignment, phylogenetic tree construction, motif, and superfamily distribution have been analyzed using various bioinformatics tools.

## 2. Material and Methods

Representative genes from histidine acid phytases (*E. coli* AppA, GenBank accession number P07102; *Aspergillus niger *PhyA and PhyB, P34752 and P34754) were used as probes to BLAST microbial genome database from NCBI (http://www.ncbi.nlm.nih.gov/). The protein sequences in FASTA format from RefSeq entries, which were shown to exhibit phytase activities, were selected for further* in silico* study. 

Physiochemical data were generated from various tools in the EXPASY proteomic server (ClustalW, ProtParam, protein calculator, Compute pI/Mw, ProtScale) [29]. The molecular weights (kDa) of the various histidine acid phytases were calculated by the addition of average isotopic masses of amino acid in the protein and deducting the average isotopic mass of one water molecule. The pI of enzyme was calculated using pK values of amino acid according to Bjellqvist et al. [30]. 

The evolutionary history was inferred using the Neighbor-Joining method [31]. The tree is drawn to scale, with branch lengths in the same units as those of the evolutionary distances used to infer the phylogenetic tree. The evolutionary distances were computed using the Poisson correction method [32] and are in the units of the number of amino acid substitutions per site. All positions containing gaps and missing data were eliminated. There were a total of 303 positions in the final dataset. Evolutionary analyses were conducted in MEGA5 [33]. For domain search, the Pfam site (http://www.sanger.ac.uk/resources/software/) was used. Domain analysis was done using MEME (http://meme.nbcr.net/meme/) [34]. The conserved protein motifs deduced by MEME were characterized for biological function analysis using protein BLAST, and domains were studied with InterProScan providing the best possible match based on the highest similarity score.

## 3. Result and Discussion 

The 50 protein sequences of HAPhy were retrieved from NCBI. The accession number of retrieved sequences along with species names is listed in Table 1. The sequences were characterized for homology search, multiple sequences alignment, biochemical features, phylogenetic tree construction, motifs, and superfamily search using various bioinformatics tools. Out of 50 sequences 12 sequences belong to HAPhy gene AppA, 26 sequences to PhyA, and 12 sequences to PhyB.

Multiple sequence alignment showed presence of conserved sites for HAPhy N-terminal “RHG/NXRXP” and C-terminal “HD” in all sequences as reported by other coworkers [25]. This is consistent with Pfam analysis of predicted active site residues, which in all sequences is shown to be N-terminal histidine residue present in conserved region and C-terminal aspartic acid. The histidine in N-terminal region seems as a nucleophile in the formation of a covalent phosphohistidine intermediate [35]. Aspartic acid at C-terminal “HD” sequence acts as a proton donor to the oxygen atom of the scissile phosphomonoester bond [36, 37]. No conserved sequence representing 3- or 6-phytase could be identified using multiple-sequence alignment.

**Figure 1:**
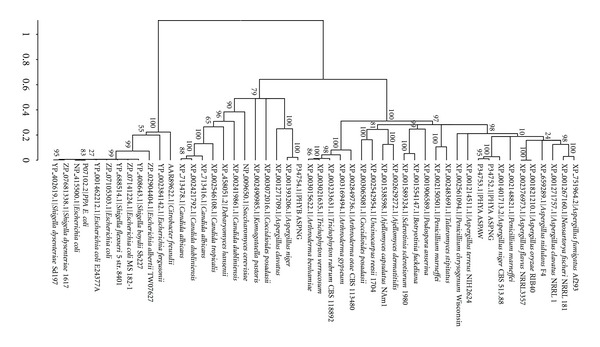
Phylogenetic tree constructed by NJ method based on HAPhy protein sequences.

The phylogenetic tree based on protein sequences revealed three major clusters. Cluster 1, a larger cluster containing 26 sequences under study, includes the majority of *Aspergillus* sp., *Penicillium* sp., *Ajellomyces* sp., *Arthroderma* sp., *Trichophyton* sp., *Sclerotinia* sp., *Uncinocarpus* sp., and *Coccidioides* sp. (Figure 1). Biochemical features for this cluster are listed in Table 2. The total number of amino acid residues ranged from 441 to 539 with variable molecular weights. pI values of this cluster ranged from 4.87 to 8.53. Variations among various phytase in this group in terms of other physiochemical parameters like positively charged and negatively charged residues, hydropathicity (GRAVY) are given in Table 2.

Aliphatic index analysis reveals uniformity in this group of phytases within the range of 75 ± 5 except for some sequences of *Arthroderma *sp. (XP_002849736.1, XP_003169494.1, XP_003015622.1) and *Trichophyton *sp. (XP_003021635.1). Aliphatic index of protein measures the relative volume occupied by aliphatic side chains of the amino acids: alanine, valine, leucine, and isoleucine. Globular proteins with high aliphatic index have high thermostability, and an increase in aliphatic index increases protein thermostability [38, 39].

Cluster 2 includes 12 protein sequences and represents PhyB gene sequences including the majority of *Candida *sp*., S. cerevisiae, C. posadasii*, and* D. hansenii*. Total number of sequences in this group is in the range of 457 to 479, and the pI values range from 4.41 to 5.82. It has less variation in its pI as compared to cluster 1 sequences (PhyA). Aliphatic index of this cluster sequences is uniform in the range of 75 ± 5 except for *Candida tropicalis* (XP_002546108.1) with a value of 67.74 and *Komagataella pastoris* (XP_002490985.1) with a value of 84.19.

Cluster 3 represents protein sequences from phytase gene AppA, also abbreviated as PhyC [22], which includes *E. coli* (in majority) along with various *Shigella *sp. and *Citrobacter freundii*. Various biophysical parameters for this group of sequences reveal amino acid residues ranging from 428 to 523, while pI value of the majority of sequences is in range of 5.5 to 6.5 except for *E. albertii *(9.35) and *E. fergusonii* (8.37). Aliphatic index of this group of sequences reveals highest thermostability among all three clusters. Predominantly positively charged amino acids are present in all three clusters.

The instability index is used to measure *in vivo* half-life of a protein [40]. The proteins which have been reported as *in vivo* half-life of less than 5 hours showed instability index greater than 40, whereas those having more than 16 hours half-life [41] have an instability index of less than 40. Instability index of HAP sequences under the study is found higher than 40 (Table 2) for 15 sequences including fully characterized *E. coli* and *A. niger* phytases, indicating an *in vivo *half-life of less than 5 hours. Superfam tool on ExPASy server for superfamily analysis of phytase sequences reveals the identity of all sequences to histidine acid phosphatase family belonging to phosphoglycerate mutase-like superfamily [42] (Table 3).

Histidine acid phytase from all three clusters shares a large *α*/*β* and a small *α*-domain [22]. MEME analysis results in frequently observed 10 motifs (Table 4). A set of 41 amino acid residues “SPFCDLFTHEEWIQYDYLQSLGKYYGYGAGNPLGPAQGIGF” representing motif 1 were conserved and uniformly observed in 38 phytase protein sequences from clusters 1 and 2, that is, PhyA and PhyB, revealing their identity with HP_HAP like, histidine acid phosphatase superfamily. Other motifs are associated with HAP superfamily (Table 2). Cluster 3, representing AppA, does not have motif 1 in its sequences, but it does contain a 50 amino acid residues long unique motif 9 “KKGCPQSGQVAI IADVDERTRKTGEAFAAGLAPDCAITVHTQADTSSPDP.” Motif 5 “YAFLKTYNYSL GADDLTPFGEQQLVDSGIKFYQRYESLAKDIVPFIRASG” is present in all protein sequences representing PhyA cluster 1. PhyB protein sequences also contain a unique 41 amino acid residues long motif 8 “ETSPENSEGPYAGTTNALRHGAAFRARY GSLYDENSTLPVF.”

## 4. Conclusion

Phylogenetic clustering and variation among biochemical features of different phytases might contribute in further classification of highly diverse HAPhys and their selection for various application purposes. Conserved sequences in motifs may be utilized for designing specific degenerate primers for identification and isolation of type and class of phytase (HAPhy) as numerous phytases are being isolated to fulfill the need of efficient phytase for feed application in various systems. Variation in biochemical features may be a key source of information for the screening of novel phytases and comparison with other classes of phytases. Functional attributes are needed to verify experimentally for conserved motifs found. This *in silico* analysis might be used for future genetic engineering of industrially important phytase.

## Figures and Tables

**Table 1:** List of retrieved protein sequences from NCBI/Entrez and their accession number.

S. no.	Source organism	Accession number	Total sequences
1	*Escherichia coli *	P07102.2, NP_415500.1, ZP_07105303.1, YP_001462212.1	4
2	*Shigella boydii *	YP_408643.1	1
3	*Shigella flexneri *	YP_688514.1	1
4	*Shigella dysenteriae *	ZP_07681338.1, YP_402619.1	2
5	*Escherichia albertii *	ZP_02904404.1	1
6	*Escherichia fergusonii *	YP_002384142.1	1
9	*Citrobacter freundii *	AAR89622.1	1
10	*Aspergillus niger *	P34752.1, XP_001401713.2, P34754.1, XP_001393206.1	4
11	*Aspergillus oryzae *	XP_001821210.1	1
12	*Aspergillus awamori *	P34753.1	1
13	*Aspergillus flavus *	XP_002376973.1	1
14	*Aspergillus fumigates *	XP_751964.2	1
15	*Aspergillus terreus *	XP_001214511.1	1
16	*Neosartorya fischeri *	XP_001267160.1	1
17	*Aspergillus nidulans *	XP_659289.1	1
18	*Aspergillus clavatus *	XP_001271757.1, XP_001271709.1	2
19	*Penicillium chrysogenum *	XP_002561094.1	1
20	*Penicillium marneffei *	XP_002148821.1	1
21	*Ajellomyces dermatitidis *	XP_002629272.1	1
22	*Botryotinia fuckeliana *	XP_001554147.1	1
23	*Uncinocarpus reesii *	XP_002542954.1	1
24	*Ajellomyces capsulatus *	XP_001538598.1	1
25	*Sclerotinia sclerotiorum *	XP_001589324.1	1
26	*Coccidioides posadasii *	XP_003065081.1	1
27	*Trichophyton rubrum *	XP_003233631.1	1
28	*Arthroderma otae *	XP_002849736.1	1
29	*Talaromyces stipitatus *	XP_002483691.1	1
30	*Podospora anserina *	XP_001906589.1	1
31	*Trichophyton verrucosum *	XP_003021635.1	1
32	*Arthroderma gypseum *	XP_003169494.1	1
33	*Penicillium marneffei *	XP_002150501.1	1
34	*Arthroderma benhamiae *	XP_003015622.1	1
35	*Candida albicans *	XP_713416.1	1
36	*Candida dubliniensis *	XP_002421792.1, XP_002419861.1	2
37	*Candida albicans *	XP_713478.1	1
38	*Candida tropicalis *	XP_002546108.1	1
39	*Debaryomyces hansenii *	XP_458051.2	1
40	*Komagataella pastoris *	XP_002490985.1	1
41	*Saccharomyces cerevisiae *	NP_009650.1	1
42	*Coccidioides posadasii *	XP_003072016.1	1

**Table 2:** Biochemical characteristics of HAPhy protein sequences.

S. no.	Accession number	Source organisms	Number of amino acids	Molecular weight	Theoretical pI	Total number of negatively charged residues (Asp + Glu)	Total number of positively charged residues (Arg + Lys)	Instability index	Aliphatic index	GRAVY	Predictive active sites by Pfam
1	P07102.2	*Escherichia coli *	523	56118.9	6.07	51	43	45.95	86.25	−0.221	39(H), 326(D)
2	NP_415500.1	*Escherichia coli str. K-12 *	432	47056.8	6.26	40	37	38.08	93.08	−0.157	39(H), 326(D)
3	ZP_07105303.1	*Escherichia coli MS 119-7 *	442	48081	6.09	41	37	39.71	92.08	−0.147	49(H), 336(D)
4	YP_001462212.1	*Escherichia coli E24377A *	432	47029.8	6.09	40	36	38.56	93.31	−0.138	39(H), 326(D)
5	ZP_07141224.1	*Escherichia coli MS 182-1 *	442	48081	6.26	41	38	39.52	92.08	−0.148	49(H), 336(D)
6	YP_408643.1	*Shigella boydii Sb227 *	432	47063.8	6.09	40	36	37.99	92.41	−0.141	39(H), 326(D)
7	YP_688514.1	*Shigella flexneri *	432	47105.9	5.94	40	35	38.2	92.87	−0.131	39(H), 326(D)
8	ZP_07681338.1	*Shigella dysenteriae 1617 *	434	47354.1	5.55	42	34	38.57	93.99	−0.142	41(H), 328(D)
9	YP_402619.1	*Shigella dysenteriae Sd197 *	434	47328	5.55	42	34	38.57	93.09	−0.152	41(H), 328(D)
10	ZP_02904404.1	*Escherichia albertii *	439	48000.6	9.35	33	44	37.78	96.88	−0.094	46(H), 333(D)
11	YP_002384142.1	*Escherichia fergusonii *	428	46608.5	8.37	32	35	40.79	93.48	−0.132	39(H), 322(D)
12	AAR89622.1	*Citrobacter freundii *	433	48506.5	6.29	49	47	35.09	86	−0.322	39(H), 325(D)
13	P34752.1	*Aspergillus niger *	467	51086	4.94	51	34	44.72	76.62	−0.211	82(H), 382(D)
14	P34753.1	*Aspergillus awamori *	467	51074.9	4.89	52	33	42.73	76.85	−0.221	82(H), 382(D)
15	XP_001401713.2	*Aspergillus niger *	497	54579.1	5.25	53	38	44.74	77.69	−0.225	112(H), 392(D)
16	XP_001821210.1	*Aspergillus oryzae *	466	51257.1	4.87	57	39	34.89	70.49	−0.316	81(H), 361(D)
17	XP_002376973.1	*Aspergillus flavus *	496	54729.2	5.11	60	46	35.72	72.7	−0.31	111(H), 391(D)
18	XP_751964.2	*Aspergillus fumigates *	498	54538.8	8.53	48	53	29.31	77.59	−0.197	114(H), 393(D)
19	XP_001214511.1	*Aspergillus terreus *	466	51088.1	5.12	51	33	35.3	72.94	−0.226	82(H), 382(D)
20	XP_001267160.1	*Neosartorya fischeri *	464	50787.1	6.17	47	43	31.33	73.17	−0.206	80(H), 359(D)
21	XP_659289.1	*Aspergillus nidulans *	463	51816.2	5.35	52	39	32.09	72.48	−0.287	80(H), 358(D)
22	XP_001271757.1	*Aspergillus clavatus *	465	51531.3	7.14	52	52	29.94	72.6	−0.332	81(H), 360(D)
23	XP_002561094.1	*Penicillium chrysogenum *	483	53668.6	7.11	51	51	46.27	69.65	−0.393	96(H), 378(D)
24	XP_002148821.1	*Penicillium marneffei *	465	50878	5.19	48	37	32.37	73.46	−0.172	80(H), 360(D)
25	XP_002629272.1	*Ajellomyces dermatitidis *	528	58565.2	6.21	55	49	44	80.49	−0.167	140(H), 420(D)
26	XP_001554147.1	*Botryotinia fuckeliana *	529	57902.2	5.08	55	42	40.6	69.38	−0.329	140(H), 423(D)
27	XP_002542954.1	*Uncinocarpus reesii *	501	56117.4	8.51	52	57	33.68	70.08	−0.448	108(H), 388(D)
28	XP_001538598.1	*Ajellomyces capsulatus *	441	49342.6	5.88	46	35	44.48	79.59	−0.238	53(H), 333(D)
29	XP_001589324.1	*Sclerotinia sclerotiorum *	465	50709.3	4.88	45	29	38.75	73.25	−0.188	76(H), 359(D)
30	XP_003065081.1	*Coccidioides posadasii *	539	60239.3	7.93	61	63	29.66	75.25	−0.368	145(H), 425(D)
31	XP_003233631.1	*Trichophyton rubrum *	474	52142.7	6.23	54	50	41.86	69.22	−0.331	87(H), 362(D)
32	XP_002849736.1	*Arthroderma otae *	466	51429.8	5.58	53	45	40.37	67.04	−0.33	85(H), 360(D)
33	XP_002483691.1	*Talaromyces stipitatus *	523	58182.2	4.9	65	42	42.55	78.7	−0.26	129(H), 409(D)
34	XP_001906589.1	*Podospora anserina *	514	57367.6	5.55	63	53	36.91	77.8	−0.396	125(H), 406(D)
35	XP_003021635.1	*Trichophyton verrucosum *	456	50332.6	6.27	53	49	41.1	66.36	−0.372	69(H), 344(D)
36	XP_003169494.1	*Arthroderma gypseum *	473	52338.9	6.33	54	51	38.25	67.06	−0.353	86(H), 361(D)
37	XP_002150501.1	*Penicillium marneffei *	510	57067.8	5.07	64	45	40.04	74.18	−0.345	116(H), 396(D)
38	XP_003015622.1	*Arthroderma benhamiae *	456	50490.7	6.14	54	49	43.2	65.72	−0.4	69(H), 344(D)
39	P34754.1	*Aspergillus niger *	479	52611.5	4.65	48	28	34.04	71.96	−0.279	82(H), 382(D)
40	XP_001393206.1	*Aspergillus niger *	479	52486.2	4.62	49	27	33.71	71.17	−0.289	82(H), 382(D)
41	XP_001271709.1	*Aspergillus clavatus *	460	50746.9	4.61	54	31	39.64	79.96	−0.17	69(H), 329(D)
42	XP_713416.1	*Candida albicans *	461	51283.1	5.8	48	43	29.87	74.27	−0.44	73(H), 335(D)
43	XP_002421792.1	*Candida dubliniensis *	462	51275.9	5.44	48	41	28.02	72.62	−0.411	73(H), 335(D)
44	XP_713478.1	*Candida albicans *	462	51305	5.57	48	42	27.7	71.99	−0.426	73(H), 335(D)
45	XP_002546108.1	*Candida tropicalis *	465	52540.6	4.41	67	35	33.65	67.74	−0.543	73(H), 337(D)
46	XP_458051.2	*Debaryomyces hansenii *	464	51835.7	5.13	52	37	39.65	73.1	−0.404	73(H), 337(D)
47	XP_002419861.1	*Candida dubliniensis *	457	52259.6	5.2	57	46	36.41	70.55	−0.493	73(H), 332(D)
48	XP_002490985.1	*Komagataella pastoris *	468	52690.7	4.41	68	33	37.25	84.19	−0.27	84(H), 346(D)
49	NP_009650.1	*Saccharomyces cerevisiae *	467	52776.5	4.43	67	36	30.82	71.46	−0.373	75(H), 338(D)
50	XP_003072016.1	*Coccidioides posadasii *	403	45575.2	5.82	51	42	31.24	70.97	−0.473	4(H), 269(D)

**Table 3:** Distribution of superfamily among HAPhy protein sequences determined using superfam server.

Family	Superfamily	Accession number (range of amino acids residues)
Histidine acid phosphatase	Phosphoglycerate mutase-like	XP_001401713.2 (61–496), P07102.2 (26–429), NP_415500.1 (26–429), ZP_07105303.1 (36–439), YP_001462212.1 (26–429), ZP_07141224.1 (36–439), YP_408643.1 (26–429), YP_688514.1 (26–429), ZP_07681338.1 (28–431), YP_402619.1 (28–431), ZP_02904404.1 (33–436) YP_002384142.1 (27–424), AAR89622.1 (27–427), P34752.1 (30–466), P34753.1 (31–466), XP_001821210.1 (29–465), XP_002376973.1 (59–495), XP_751964.2 (62–497), XP_001214511.1 (32–466), XP_001267160.1 (28–463), XP_659289.1 (28–461), XP_001271757.1 (30–464), XP_002561094.1 (45–482), XP_002148821.1 (28–464), XP_002629272.1 (89–527), XP_001554147.1 (90–527), XP_002542954.1 (57–495), XP_001538598.1 (6–440), XP_001589324.1 (26–463), XP_003065081.1 (97–532), XP_003233631.1 (36–469), XP_002849736.1 (35–466), XP_002483691.1 (87–517), XP_001906589.1 (76–512), XP_003021635.1 (23–451), XP_003169494.1 (35–468), XP_002150501.1 (73–505), XP_003015622.1 (23–451), P34754.1 (35–470), XP_001393206.1 (35–470), XP_001271709.1 (23–452), XP_713416.1 (28–455), XP_002421792.1 (28–455), XP_713478.1 (28–455), XP_002546108.1 (28–457), XP_458051.2 (28–451), XP_002419861.1 (28–445), XP_002490985.1 (42–464), NP_009650.1 (34–460), XP_003072016.1 (1–393)

**Table 4:** Distribution of commonly observed motifs in different HAPhy protein sequences along with their functional domains.

Motifs number	Motif present in number of sequence	Motif width	Sequence	Domain
1	38	41	SPFCDLFTHEEWIQYDYLQSLGKYYGYGAGNPLGPAQGIGF	HP_HAP_like, histidine phosphatases superfamily
2	49	29	VPPGCKITFVQVLSRHGARYPTKSKSKMY	Histidine phosphatase superfamily
3	47	30	VRVLVNDRVVPLHGCLVDPLGRCKLDDFVA	Local conserved domain
4	49	29	TLYADFSHDNDMTSIFTALGLYNGTEPLS	Histidine phosphatase superfamily
5	26	50	YAFLKTYNYSLGADDLTPFGEQQLVDSGIKFYQRYESLAKDIVPFIRASG	Histidine phosphatase superfamily
6	49	29	RLNKALPGVNLTSADVVSLMDMCSFETVA	Histidine phosphatase superfamily
7	48	21	GYSAAWTVPFGARAYFEKMQC	Histidine phosphatase superfamily
8	11	50	TEIFLLQQAQGMPEPGWGRITDSHQWNTLLSLHNAQFYLLQRTPEVARSR	Local conserved domain
9	12	50	KKGCPQSGQVAIIADVDERTRKTGEAFAAGLAPDCAITVHTQADTSSPDP	Histidine phosphatase superfamily
10	9	50	TPHPPQKQAYGVTLPTSVLFIAGHDTNLANLGGALELNWTLPGQPDNTPP	Histidine phosphatase superfamily
